# Selection and Evaluation of Reference Genes for qRT-PCR in *Spodoptera frugiperda* (Lepidoptera: Noctuidae)

**DOI:** 10.3390/insects12100902

**Published:** 2021-10-03

**Authors:** Shipeng Han, Qiuju Qin, Da Wang, Yayuan Zhou, Yunzhuan He

**Affiliations:** College of Plant Protection, Hebei Agricultural University, Baoding 071000, China; hanshipeng1994@163.com (S.H.); qiujuqin@163.com (Q.Q.); wangda@hebau.edu (D.W.); 13393230627@163.com (Y.Z.)

**Keywords:** *Spodoptera frugiperda* (J. E. Smith), reference gene, normalization, gene expression

## Abstract

**Simple Summary:**

The fall armyworm *Spodoptera frugiperda* (J. E. Smith) (Lepidoptera: Noctuidae) is an extremely important omnivorous agricultural pest, it poses a severe threat to food security and agricultural production. Quantitative real-time PCR (qRT-PCR) is an important molecular technology widely used for expression profile analyses of various target genes. It is essential to use reference genes as the benchmark to eliminate various errors and normalize the qRT-PCR analysis. In our study, 10 reference genes were evaluated under six experimental conditions, including developmental stages, tissues, mating status, hormones, diets, and temperatures. Finally, the expression profile of the target gene *SfrOBP*1 in various tissues of *S. frugiperda* was evaluated to verify the accuracy of the results. This study will provide a preliminary evaluation of reference genes of *S. frugiperda*, which can be beneficial to the further research of functional gene expression.

**Abstract:**

As an accurate and convenient technique, the qRT-PCR is always used in the quantitative expression analysis of functional genes. Normalization of the data relies on stable reference genes. The fall armyworm *Spodoptera frugiperda* (J. E. Smith) is an important invasive and migratory pest that seriously threatens corn production around the world. In this paper, we selected 10 candidate reference genes (*18S*, *AK*, *RPL10*, *RPS24*, *28S*, *SOD*, *ATP*, *GAPDH*, *ACT*, and *a-TUB*) and determined their expression levels under different conditions (different developmental stages, various tissues, mating status, hormones, diets, and temperatures). Subsequently, the stability of reference genes was evaluated by four algorithms (Delta Ct method, geNorm, NormFinder, BestKeeper). The optimal combination of reference genes for each treatment was obtained by geNorm. Finally, the comprehensive ranks were determined by the online tool RefFinder. Results showed that the most stable reference genes were *SOD*, *RPL10*, and *RPS24* for developmental stages, *α-TUB*, *RPL10*, and *ATP* for different tissues, *AK*, *RPL10*, and *18S* for mating status, *18S* and *AK* under hormone treatment, *18S*, *RPL10*, and *SOD* under diet treatment, *RPL10*, *18S*, and *RPS24* under temperature treatment. This study confirmed recent data on a few reference genes and provided an evaluation of a number of additional reference genes of *S. frugiperda* under various conditions.

## 1. Introduction

The fall armyworm *Spodoptera frugiperda* (J. E. Smith) (Lepidoptera: Noctuidae) is indigenous to the tropical and subtropical regions of the American continent, with high reproductive capacity, a wide range of hosts, and strong migration ability [1]. Therefore, this pest seriously threatens corn production and food security around the world [2,3,4]. At present, the *S. frugiperda* has invaded 46 countries or regions of Africa and nine countries of Asia [5,6,7]. In January 2019, *S. frugiperda* was first discovered in a cornfield of Jiangcheng City, Yunnan Province, China. Subsequently, it has invaded 29 provinces and posed a severe threat to the food security and agricultural production of China [2,4,6,8].

Quantitative real-time PCR (qRT-PCR) is an important molecular technology with high sensitivity, reliability, and specificity. It is always used in gene quantitative expression analysis and transcriptome verification [9,10,11,12]. However, many factors can lead to systematic errors and affect the accuracy of qRT-PCR results, such as the integrity and purity of total RNA, the efficiency of reverse transcription and PCR program, and pipetting [13,14,15]. Therefore, it is essential to use reference genes as the benchmark to eliminate various errors and normalize the qRT-PCR analysis [16]. Several traditional reference genes are always used as the benchmark to evaluate the expression profile of function genes, such as *18S* ribosomal RNA (*18S*), alpha-tubulin (*α-TUB*), beta-actin (*β-ACT*), and glyceraldehyde-3-phosphate dehydrogenase (*GAPDH*) [9,10,17,18]. These reference genes are involved in the metabolism process of insect cells. The ideal reference genes should be highly stable in various conditions [19]. So far, the reference genes have been selected in many Lepidoptera insects, including *Spodoptera exigua* (Hübner), *Heliconius numata* (Cramer), *Mythimna separata* (Walker), *Danaus plexippus* (Linnaeus), *Bombyx mori* (Linnaeus), *Chilo suppressalis* (Walker) and *Helicoverpa armigera* (Hübner) [20,21,22,23,24,25,26]. Furthermore, Boaventura et al. (2020) evaluate the expression stability of 10 reference genes across strains, larval instars, and gut tissues of *S. frugiperda* [27]. These researches proved that the reference genes show significant various expression levels under different condition treatments, and none is suitable for all experimental treatments. Accordingly, the stability of specific reference genes for various treatments should be verified before conducting qRT-PCR reactions. However, there have been only a few studies on selecting reference genes in *S. frugiperda* under different experimental conditions.

Here, we selected 10 candidate reference genes, *18S*, *AK*, *RPL10*, *RPS24*, *28S*, *SOD*, *ATP*, *GAPDH*, *ACT*, and *a-TUB*, and evaluated their stability under different treatments (including developmental stages, tissues, mating, hormones, diets, temperature) by Delta Ct method, geNorm, NormFinder, BestKeeper, and RefFinder. These findings will improve the accuracy of gene expression analysis, which can help to form a solid foundation for future research of the functional genes of *S. frugiperda*.

## 2. Materials and Methods

### 2.1. Insect Rearing

*Spodoptera frugiperda* (J. E. Smith) samples were obtained from Henan Academy of Agricultural Sciences, Zhengzhou, China. The larvae were fed with corn seedlings, and adults were reared with 10% sucrose solution. The rearing conditions were as follows, 26 ± 1 °C, 65% ± 5% relative humidity, and 16 h light and 8 h dark photoperiods.

### 2.2. Experimental Treatments and Sample Collection

#### 2.2.1. Different Developmental Stages

In this study, eggs (20 mg), first-third-instar larvae (50, 20, 5 individuals, respectively), fourth-sixth instar larvae (1 individual), third-day pupae (1 male and female individual), third-day adults (1 male and female individual) were collected as samples of different developmental stages. There has a longitudinal crack with a tumor on each side of the border in the ventral side of the 9th abdominal segment of male pupae. For female pupae, there has a longitudinal crack in the ventral side with flat sides and no humps in the 8th abdominal segment.

#### 2.2.2. Various Tissues

Third-day-old adults were dissected in pre-cooled PBS solution to collect each tissue, including heads (10 individuals), thoraxes (5 individuals), abdomens (5 individuals), legs (20 individuals), wings (20 individuals) of males; and heads (10 individuals), thoraxes (5 individuals), ovaries (5 individuals), fat body (20 mg), legs (20 individuals), wings (20 individuals) of females.

#### 2.2.3. Mating Status

To investigate the effect of mating status, mated adults of male and female (1 individual) and unmated adults of male and female (1 individual) were collected, respectively.

#### 2.2.4. Hormone Treatment

The juvenile hormone (Sigma, St. Louis, MO, USA) was dissolved in acetone (Fuyu, Tianjin, China) solution (1 μg/μL), then 1 μL juvenile hormone solution and acetone solution were injected into newly emerged female adults used microinjector (Hamilton, Bonaduz, CH), separately. Samples were collected at 6, 12, and 24 h after injection, respectively.

#### 2.2.5. Diet Treatment

*Spodoptera frugiperda* were reared with corn seedlings, rice seedlings, wheat seedlings, and artificial feed, respectively. Then third-instar larvae (5 individuals), third-day male and female pupa (1 individual), third-day male and female adult (1 individual) were collected in each condition.

#### 2.2.6. Temperature Treatment

For temperature treatment, *S. frugiperda* were reared at 20, 26, and 32 °C, respectively. Then third-instar larvae (5 individuals), third-day male and female pupae (1 individual), third-day male and female adult (1 individual) were collected in each temperature treatment.

The sample of each treatment was collected in 1.5 mL centrifuge tubes and rapidly frozen in liquid nitrogen, then stored at −80 °C. All treatments were set to three biological replicates.

### 2.3. RNA Isolation and cDNA Synthesis

According to the instruction, the total RNA of all samples was extracted by the RNAprep Pure Tissue Kit (Tiangen, Beijing, China). The RNA concentration and purity were detected with a NanoDrop spectrophotometer (MD2000C; Biofuture, UK). Subsequently, each sample of 1 μg total RNA was used for cDNA synthesis with PrimeScript RT Reagent Kit (Takara, Dalian, China).

### 2.4. Reference Genes Selection and Primer Design

Ten commonly used reference genes (*18S*, *AK*, *RPL10*, *RPS24*, *28S*, *SOD*, *ATP*, *GAPDH*, *ACT*, and *a-TUB*) were selected. The primer of each gene was designed by DNAMAN V6. The purified PCR product was used as a starting template to draw the standard curve to determine the amplification efficiency of primer, and each gradient was diluted 10-fold, with 5 gradients. The melting curve had only one single peak determined that the primer was specific.

### 2.5. qRT-PCR Analysis

The qRT-PCR amplification was performed by C1000 Touch Thermal Cycler (Bio-Rad, Hercules, CA, USA). A total of 20 μL reaction volume containing 10 μL Fast Super EvaGreen^®^ Master Mix (US Everbright Inc., Suzhou, China), 1 μL cDNA of a sample, 0.5 μL of each primer (10 μM), and 8 μL ddH_2_O. The PCR reaction conditions were as follows: 95 °C for 2 min, and 40 cycles of 95 °C for 5 s, 56 °C for 30 s, 72 °C for 30 s. The melting curves of amplicons were determined by taking continuous fluorescence readings with increasing temperatures from 65 to 95 °C. Three biological repeats were set for each reaction, and three technical repeats were set for each biological repeat.

### 2.6. Stability Analysis

The Delta Ct method [11], geNorm [28], NormFinder [15], and BestKeeper [29] were used to evaluate the stability of each reference gene in different treatments. Based on the relative pairwise comparisons of genes within each sample, the Delta Ct method can identify the most stable reference gene. geNorm is used to calculate the M value to evaluate the expression stability of reference genes. Furthermore, the geNorm can define the optimal number of reference genes by calculated pairwise variance values (V). When the value (Vn/Vn+1) is less than 0.15, it means that the optimal number is n. NormFinder is used to calculate the changes of intergroup and intragroup to determine the optimal reference gene. The BestKeeper focuses on the standard deviation and variation coefficient of Ct value, which can rank the stability of each gene. Finally, the online tool RefFinder can determine the comprehensive ranking of reference genes by integrating four algorithms.

### 2.7. Stability Verification of Candidate Reference Genes

*Odorant-binding proteins1 (SfruOBP1)* was selected to verify the stability of reference genes. The relative expression of *SfruOBP1* in tissues of *S. frugiperda* was calculated by the 2^-^^∆∆Ct^ method, using the most stable and unstable reference gene, respectively [30]. The significant differences were using IBM SPSS Statistics version 22 using one-way ANOVA analysis followed by Tukey’s test.

## 3. Results

### 3.1. qRT-PCR Analysis

Before evaluating the suitability of the reference genes, the specificity and efficiency of PCR amplification should be first confirmed. In this study, all the PCR products amplified were detected with 0.8% agarose gel, and the single band was observed in each PCR product with the expected band size. In addition, the PCR amplification for each primer pair showed a single peak in melting curves (Figure S1). The amplification efficiency of each primer pair ranged from 90.6% to 107.1%, and all regression coefficients were higher than 0.990 (Table 1). Taken together, the primers can be used for quantitative determination.

**Figure 1:**
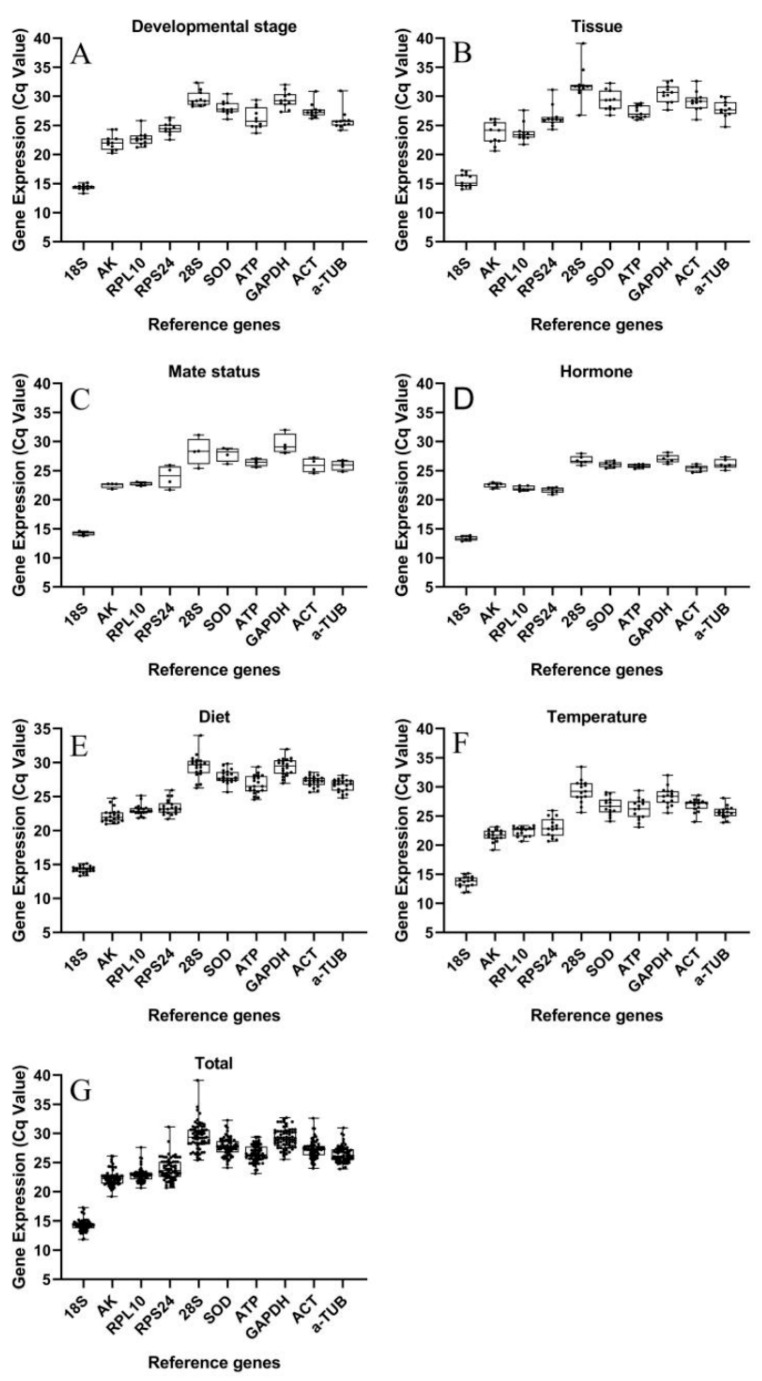
Expression profiles of 10 candidate reference genes of *S. frugiperda* in different treatments. (**A**) different developmental stages (n = 33); (**B**) various tissues (n = 33); (**C**) mating status (n = 12); (**D**) hormone treatment (n = 18); (**E**) diets treatment (n = 60); (**F**) temperature treatment (n = 45); (**G**) all samples (n = 201); the Ct values indicate the expression levels of reference genes and n represents the number of samples used to derive values; the box represents the 25th to 75th percentiles and the line in the box represents the median, and the bars represents the minimum and maximum.

We determined the Ct value of 10 candidate reference genes for various treatments, i.e., different developmental stages (Figure 1A), various tissues (Figure 1B), mating status (Figure 1C), hormone treatment (Figure 1D), diet treatment (Figure 1E), and temperature treatment (Figure 1F). The Ct values ranged from 11.85 (18S) to 39.12 (28S), and the Ct values of 18S were the lowest (11.85–17.25), and the 28S were the highest (25.39–39.12). The variation of Ct values in 18S was minimum (5.40), and the 28S had the maximum Ct value variation (13.73) (Figure 1G).

### 3.2. Expression Stability Analysis of Candidate Reference Genes

**Figure 2:**
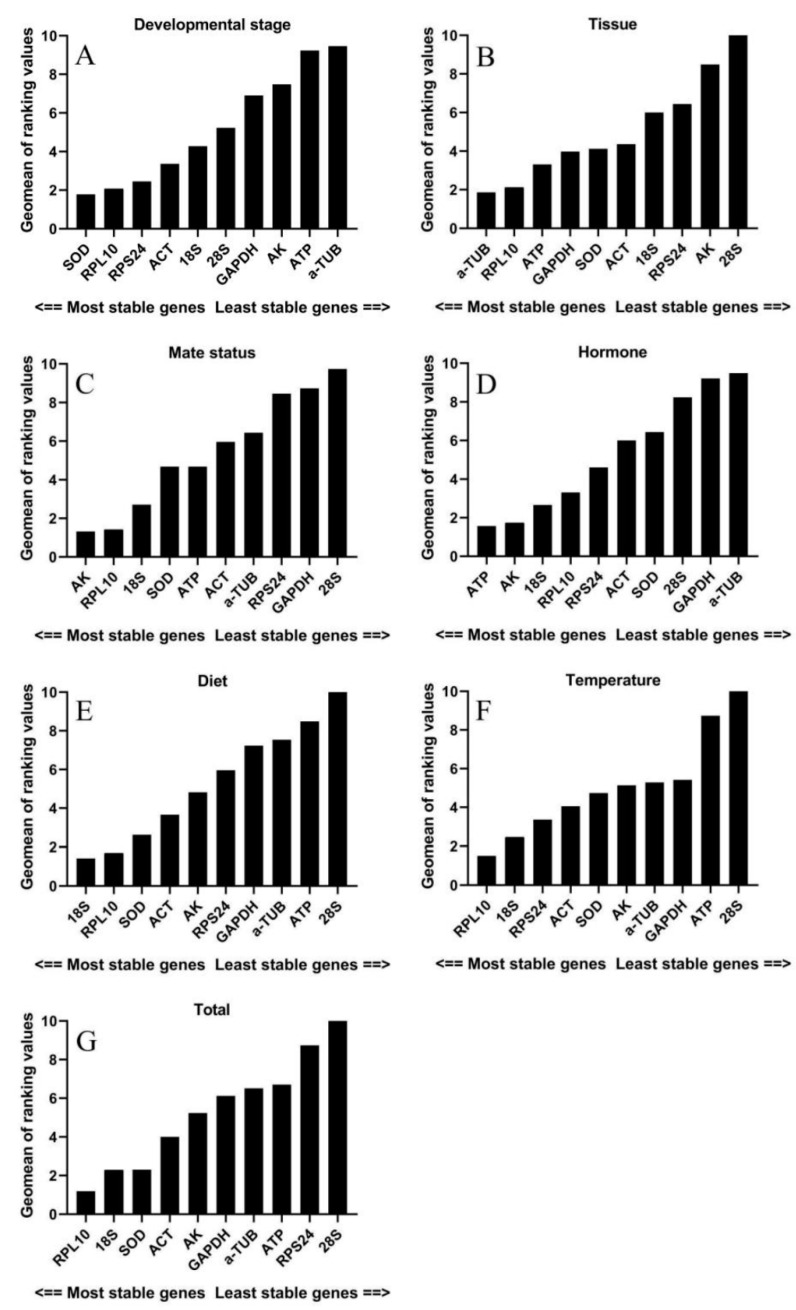
Expression stability of 10 candidate reference genes of *S. frugiperda* in different treatments by RefFinder. A lower Geomean value indicates more stable expression. (**A**) different developmental stages; (**B**) various tissues; (**C**) mating status; (**D**) hormone treatment; (**E**) diets treatment; (**F**) temperature treatment; (**G**) all samples.

**Figure 3:**
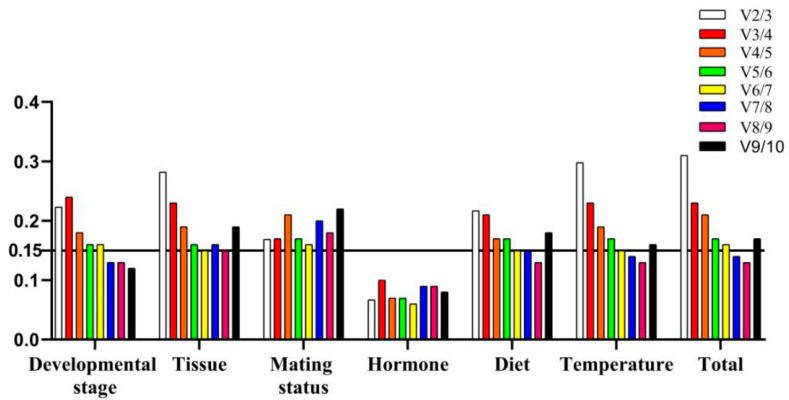
Evaluation of the optimal number of reference genes for normalization of *S. frugiperda* in different treatments. The dashed line indicates that the pairwise variation is 0.15.

For different developmental stages, the results of the Delta Ct method, geNorm, and NormFinder analysis indicated that *SOD*, *RPL10*, and *RPS24* were the most stable gene. The BestKeeper analysis identified *18S* as the most suitable reference genes (Table 2). Following the RefFinder analysis, the overall ranking of expression stability was as follow *SOD* > *RPL10* > *RPS24* > *ACT* > *18S* > *28S* > *GAPDH* > *AK* > *ATP* > *α-TUB* (Figure 2A). Moreover, geNorm analysis indicated that V7/V8 was less than 0.15, but based on the geNorm instructions, the top three genes were regarded as the optimal reference genes combination (*SOD*, *RPL10*, *RPS24*) (Figure 3).

*RPL10* was the most stable gene based on the Delta Ct method and NormFinder in various tissues. The estimation of geNorm showed that *α-TUB* and *GAPDH* were the most stable genes. However, the expression stability of *ATP* was highest in BestKeeper. Meanwhile, *28S* was regarded as the most unstable gene based on all algorithms (Table 2). Combining four algorithms, the comprehensive ranking by RefFinder was as follow *α-TUB* > *RPL10* > *ATP* > *GAPDH* > *SOD* > *ACT* > *18S* > *RPS24* > *AK* > *28S* (Figure 2B). In addition, all pairwise variance values were higher than 0.15 by geNorm analysis. Thus, *α-TUB*, *RPL10*, *ATP* were the optimal combination of reference genes (Figure 3).

Based on the results of the Delta Ct method, geNorm, and NormFinder, *AK* was identified as the most stable gene in mating status; the BestKeeper analysis showed that *RPL10* had the highest expression stability (Table 2). The RefFinder calculated comprehensive ranking was as followed *AK* > *RPL10* > *18S* > *SOD* > *ATP* > *ACT* > *α-TUB* > *RPS24* > *GAPDH* > *28S* (Figure 2C). Meanwhile, all pairwise variance values were greater than 0.15 by geNorm analysis. Therefore, the best group of reference genes for mating status were *AK*, *RPL10*, *18S* (Figure 3).

For hormone treatment, the Delta Ct method, NormFinder, and BestKeeper identified *ATP* and *AK* as the most suitable reference genes, and geNorm identified *18S* and *AK* as the most suitable reference genes (Table 3). Following the RefFinder analysis, the comprehensive ranking of expression stability was as follow *ATP* > *AK* > *18S* > *RPL10* > *RPS24* > *ACT* > *SOD* > *28S* > *GAPDH* > *α-TUB* (Figure 2D). In addition, the geNorm analysis calculated that V2/V3 was less than 0.15, indicating that two reference genes should be combined for normalization. So, *ATP* and *AK* were the optimal combinations of reference genes in hormone treatment (Figure 3).

For diet treatment, the Delta Ct method and geNorm indicated that the most stable gene was *SOD*. The expression stability of *18S* was the highest based on NormFinder and BestKeeper. Meanwhile, the expression stability of *28S* was lowest by four methods (Table 3). The RefFinder calculated comprehensive ranking of the expression stability of candidate reference genes followed as *18S* > *RPL10* > *SOD* > *ACT* > *AK* > *RPS24* > *GAPDH* > *α-TUB* > *ATP* > *28S* (Figure 2E). Moreover, the V6/V7 was smaller than 0.15 by geNorm analysis. So, the optimal combination of reference genes in diet treatment were *18S*, *RPL10*, *SOD* (Figure 3).

Based on the results of the Delta Ct method, NormFinder, and BestKeeper, *RPL10* was identified as the most stable gene in temperature treatment, whereas geNorm identified *RPS24* and *SOD* as the most suitable reference genes (Table 3). The stability comprehensive order of these genes in RefFinder analysis was listed as following *RPL10* > *18S* > *RPS24* > *ACT* > *SOD* > *AK* > *α-TUB* > *GAPDH* > *ATP* > *28S* (Figure 2F). Furthermore, geNorm analysis indicated that V7/V8 is less than 0.15, *RPL10*, *18S*, and *RPS24* were regarded as the optimal combination of reference genes for temperature treatment (Figure 3).

For all samples, *RPL10* was regarded as the most stable gene based on the Delta Ct method, geNorm, and NormFinder. The expression stability of *18S* was the highest in BestKeeper analysis. Meanwhile, the stability of *28S* was the worst in all algorithms (Table 3). Combining four algorithms, the comprehensive order by RefFinder was as follow *RPL10* > *18S* > *SOD* > *ACT* > *AK* > *GAPDH* > *α-TUB* > *ATP* > *RPS24* > *28S* (Figure 2G). In addition, the V7/V8 was smaller than 0.15 by geNorm analysis. So, the optimal combination of reference genes in all samples were *RPL10*, *18S*, *SOD* (Figure 3).

### 3.3. Verification of Candidate Reference Genes

**Figure 4:**
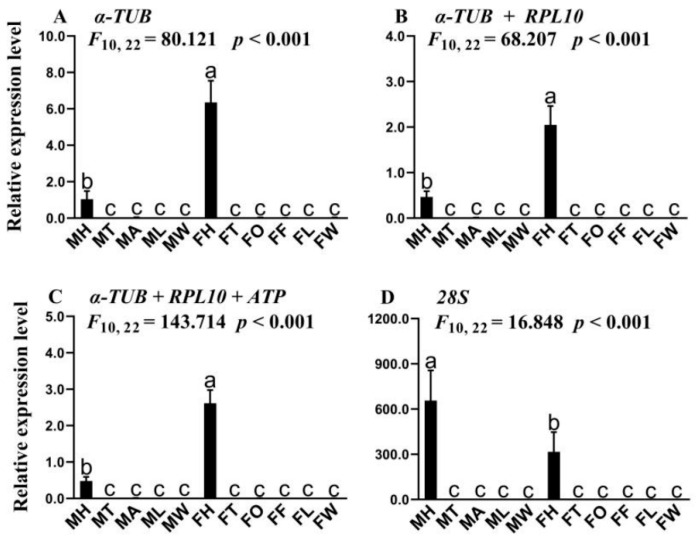
Expression level of *SfruOBP*1 in various tissues of *S. frugiperda*. MH: male head, MT: male thorax, MA: male abdomen, ML: male leg, MW: male wing; FH: female head, FT: female thorax, FO: female ovary, FF: female fat body, FL: female leg, FW: female wing. Relative expression levels of *OBP*1 were normalized with (**A**): *α-TUB* (most stable reference gene), (**B**): α-TUB and RPL10 (the two most stable reference genes), (**C**): *α-TUB*, *RPL10*, and *ATP* (optimal combination of reference genes) and (**D**): *28S* (least stable reference gene). The data in the figures are means ± SE. Means are from three biological replicates. Different letters above bars indicate significant differences (*p* < 0.05, one-way ANOVA analysis followed by Tukey’s test).

We selected *SfruOBP1* as the target gene, and four candidate genes (*α-TUB*, *RPL10*, *ATP*, *28S*) were used as reference genes to determine the expression level of the target gene in different tissues of *S. frugiperda*. When the more stable genes (*α-TUB*, *RPL10*, *ATP*) were used as the reference genes, *SfruOBP1* was significantly expressed in the head of females (Figure 4A–C). However, when the unstable gene (*28S*) was used as the reference gene, the expression level of *SfruOBP1* in the head of males was significantly higher than females (Figure 4D).

## 4. Discussion

The qRT-PCR is a reliable technique in gene expression analysis, with high sensitivity and specificity, and the qRT-PCR data must be normalized by suitable reference genes to avoid expression differences among samples [31]. Recently, there have many reports on reference genes selection of various insects under different abiotic and biotic conditions [32,33,34,35,36], including *S. frugiperda* [27]. In these studies, the expression levels of conventional reference genes in different insects are quite different, and none of them with similar expression levels under all conditions, which indicates that there is no absolute universality among homologous reference genes. Thus, screening reference genes are essential for quantitative research under certain conditions.

In this study, the Delta Ct method, geNorm, NormFinder, and BestKeeper were used to evaluate the expression stability of candidate reference genes. The results indicated that the ranks of candidate reference genes were significantly different by various algorithms. For example, the expression stability of *RPL10* was the highest in various tissues of *S. frugiperda* based on the Delta Ct method and NormFinder. Moreover, the geNorm and BestKeeper indicated that the most stable gene was *GAPDH* and *ATP*, respectively. In addition, *RPL10* was regarded as the most stable gene based on the Delta Ct method, NormFinder, and BestKeeper under hormone treatment, and the expression stability of *18S* was the highest in geNorm analysis. Similarly, following the Delta Ct method, geNorm, and NormFinder analysis, Xu et al. (2017) find that the expression stability of *EF1* is the highest of *Chilo suppressalis* (Walker) under temperature stress, and *RPS11* is the lowest, but *RPS11* is the most stable gene, and *EF1* is an unstable gene in BestKeeper analysis. These results also occur in other insects, as each program is based on its own unique algorithm [24,37]. Therefore, in order to eliminate the error of the algorithm of the four methods, the online program RefFinder was used to provide a comprehensive ranking. The results showed that *SOD* for different developmental stages, *α-TUB* for various tissues, *AK* for mating treatment, *18S* for hormone treatment, *ATP* for diets treatment, *RPL10* for temperature treatment were the most stable reference genes of *S. frugiperda*. These results also further verified that the expression stability of the reference gene varied under different treatments.

Ribosomal protein (RP) is the component of ribosomes. It plays a vital role in protein biosynthesis in all biological cells. It also participates in cell growth regulation, cell differentiation, and DNA repair [38,39]. In this paper, *RPL10* was the most stable gene in temperature treatment and all test samples of *S. frugiperda*. Meanwhile, *RPL10* also showed a relatively high stable ranking in other treatments of *S. frugiperda*. Similarly, Boaventura et al. evaluated several candidate reference genes for normalization of gene expression data across strains, larval instars, and gut tissues of *S. frugiperda* and identified that *RPL10* was a stable reference gene [27]. Previous research also indicates that ribosomal protein genes, as commonly used reference genes, have a wide range of applications in many insects. For example, *RPL27* and *RPL32* in different developmental stages and tissues of *Apolygus lucorum* (Meyer-Dur) [33], *RPL12* in starvation treatment of *Anthonomus eugenii* (Cano) [9], and *RPS20* in temperature treatment of *Diaphorina citri* (Kuwayama) [40]. However, the stability of ribosomal protein genes is not universal in all insects [24,34].

With the in-depth study of the stability of reference genes, many researchers have proposed that using multiple reference genes can improve the accuracy of qRT-PCR and remove biased normalization [15,41,42]. The geNorm analysis not only evaluates the stability of reference genes but also calculates the best combination of the reference gene under certain conditions. Based on the geNorm instructions, when pairwise variance value is less than 0.15, it means that the number of optimal combinations is n; when the pairwise variation values are greater than 0.15, we can select two or three most stable reference genes as combination according to the trend of pairwise variation value. In this paper, the pairwise variance value V2/V3 of hormone treatment was less than 0.15, so we needed two reference genes to analyze the gene expression. Although the V3/V4 of other treatments were more than 0.15, we propose that three of the best reference genes should be used to obtain accurate and reliable results under these conditions. Many studies have shown that more reference genes are needed for accurate normalization when the sample size becomes larger. However, the accuracy of target gene expression is decreased when the fourth reference gene is introduced [41,42]. It followed that the threshold of pairwise variance values (V < 0.15) is not absolute.

Odorant-binding protein is one of the most important proteins in the olfactory system of insects [43]. In this study, we determined the expression levels of *SfruOBP1* in various tissues to verify the stability of reference genes. The results indicated that the expression patterns of *SfruOBP1* were similar when the most stable and recommended combination of reference genes in various tissues were used as the benchmark, respectively; it was significantly expressed in the head of the female. However, the expression pattern of *SfruOBP1* was different from that mentioned above when the least stable reference gene was selected as the benchmark, and *SfruOBP1* was significantly expressed in the head of the male. Therefore, screening suitable reference genes are essential to improve the accuracy of function gene expression.

## 5. Conclusions

In conclusion, the stability of 10 candidate reference genes was analyzed by five reliable algorithms under different experimental conditions. The results showed that the optimal combination of most stable reference genes was *SOD*, *RPL10*, and *RPS24* for developmental stages; *α-TUB*, *RPL10*, and *ATP* for various tissues; *AK*, *RPL10*, and *18S* for mating status; *18S* and *AK* under hormone treatment; *18S*, *RPL10*, and *SOD* under diet treatment; *RPL10*, *18S*, and *RPS24* under temperature treatment. This study confirmed recent data on some reference genes and provided an evaluation of a number of additional reference genes of *S. frugiperda* under various conditions.

## Figures and Tables

**Table 1:** Primers used for qRT-PCR in *S. frugiperda*.

Gene	GenBank Accession Number	Primer Sequences (5′-3′)	Length (bp)	Amplification Efficiency (%)	Regression Coefficients
*18S*	KY554596	F: GACTCAACACGGGAAATCTC R: CCACGCACACCTAAATGAC	185	102.6	0.995
*AK*	KC262642	F: TCTACCACAACGAGAACAAGAC R: AAAGTGAGGAAACCAAGCC	180	97.88	0.992
*RPL10*	XM_035580073	F: TGGGTAAGAAGAAGGCTACG R: TGTTGATGCGGATGACAT	194	104.2	0.994
*RPS24*	XM_035577015	F: CACTGGCTTTGCTCTCATC R: TCATCCTGTTCTTGCGTTC	136	102.5	0.995
*28S*	XM_035580587	F: GACCAGATTCCGATTTCACT R: CTTTGTTTACCGCTTGCC	122	94.1	0.996
*SOD*	XM_035587671	F: TCGGCACAATCATCAGTC R: AGTCCTTCTCAATAGCCTGC	137	93.6	0.995
*ATP*	XM_035575197	F: AAAGTAGTTCCGTGGAGTGAG R: AAAGAAGGGTCCGTAGATTG	115	107.1	0.994
*GAPDH*	XM_035587881	F: AGAAGACTGTTGACGGACC R: AGGAATGACTTTGCCGAC	112	103.9	0.984
*ACT*	KT218672	F: TTCTTCCACCCTGAGTTCTC R: AGTCCTCTTGATGTCACGC	182	90.6	0.999
*a-TUB*	XM_035600854	F: AGGGCTGTGTTTGTTGACT R: TCCTTACCGATGGTGTAGTG	148	93.6	0.999
*OBP*1	XM_035578840	F: GTGGGTGTGAGAGAGATAGAAC R: GAACAGGTCTGCTATGATGTG	125	100.0	0.994

**Table 2:** Expression stability of 10 candidate reference genes of *S. frugiperda* in different developmental stages, tissues, and mating status.

Treatments	Genes	Delta Ct		geNorm		NormFinder		BestKeeper	
		Stability	Ranking	Stability	Ranking	Stability	Ranking	Stability	Ranking
Developmental stages	*18S*	1.282	9	0.954	6	0.680	7	0.352	1
	*AK*	1.236	7	1.081	8	0.683	8	1.053	7
	*RPL10*	0.885	2	0.607	3	0.174	1	0.822	3
	*RPS24*	1.061	3	0.444	1	0.453	3	0.825	4
	*28S*	1.117	5	0.873	5	0.625	5	1.010	6
	*SOD*	0.813	1	0.444	1	0.189	2	0.904	5
	*ATP*	1.255	8	1.143	9	0.803	9	1.498	10
	*GAPDH*	1.207	6	1.037	7	0.677	6	1.104	9
	*ACT*	1.065	4	0.797	4	0.557	4	0.807	2
	*a-TUB*	1.283	10	1.197	10	0.820	10	1.079	8
Tissues	*18S*	1.649	9	1.168	8	0.961	9	1.043	2
	*AK*	1.563	8	1.251	9	0.937	8	1.694	9
	*RPL10*	1.058	1	0.995	5	0.318	1	1.084	4
	*RPS24*	1.209	6	1.073	7	0.580	5	1.246	7
	*28S*	1.964	10	1.405	10	1.284	10	1.752	10
	*SOD*	1.142	3	0.802	3	0.502	4	1.374	8
	*ATP*	1.153	5	0.914	4	0.587	6	0.919	1
	*GAPDH*	1.228	7	0.625	1	0.593	7	1.176	6
	*ACT*	1.146	4	1.036	6	0.499	3	1.135	5
	*a-TUB*	1.092	2	0.625	1	0.483	2	1.080	3
Mating status	*18S*	1.182	4	0.408	3	0.388	3	0.268	2
	*AK*	1.062	1	0.190	1	0.066	1	0.338	3
	*RPL10*	1.067	2	0.190	1	0.181	2	0.212	1
	*RPS24*	1.703	8	1.177	8	1.069	8	1.568	10
	*28S*	1.968	10	1.516	10	1.496	10	1.459	9
	*SOD*	1.182	4	0.770	5	0.416	4	0.913	6
	*ATP*	1.098	3	0.556	4	0.644	6	0.485	4
	*GAPDH*	1.765	9	1.312	9	1.204	9	1.220	8
	*ACT*	1.185	6	0.896	6	0.549	5	1.028	7
	*a-TUB*	1.401	7	0.999	7	0.846	7	0.633	5

**Table 3:** Expression stability of 10 candidate reference genes of *S. frugiperda* under hormone treatment, diet treatment, and temperature treatment.

Treatments	Genes	Delta Ct		geNorm		NormFinder		BestKeeper	
		Stability	Ranking	Stability	Ranking	Stability	Ranking	Stability	Ranking
Hormone treatment	*18S*	0.545	7	0.210	1	0.216	5	0.316	3
	*AK*	0.484	2	0.210	1	0.119	2	0.315	2
	*RPL10*	0.502	4	0.341	5	0.119	2	0.338	4
	*RPS24*	0.506	5	0.316	4	0.162	4	0.436	7
	*28S*	0.675	8	0.486	8	0.499	8	0.554	9
	*SOD*	0.509	6	0.409	7	0.271	6	0.413	5
	*ATP*	0.432	1	0.222	3	0.106	1	0.267	1
	*GAPDH*	0.799	10	0.631	10	0.557	9	0.541	8
	*ACT*	0.499	3	0.582	6	0.271	6	0.419	6
	*a-TUB*	0.703	9	0.563	9	0.565	10	0.629	10
Diet treatment	*18S*	1.041	4	0.808	3	0.224	1	0.385	1
	*AK*	1.230	6	0.694	5	0.654	6	0.787	6
	*RPL10*	1.034	3	0.636	1	0.363	2	0.562	2
	*RPS24*	1.254	8	0.957	5	0.652	5	0.812	7
	*28S*	1.713	10	1.319	10	1.221	10	1.239	10
	*SOD*	0.958	1	0.636	1	0.441	4	0.751	4
	*ATP*	1.249	7	1.165	9	0.745	8	1.149	9
	*GAPDH*	1.256	9	1.024	7	0.715	7	1.055	8
	*ACT*	1.003	2	0.870	4	0.416	3	0.596	3
	*a-TUB*	1.214	5	1.100	8	0.793	9	0.763	5
Temperature treatment	*18S*	1.127	3	0.950	3	0.393	2	0.810	3
	*AK*	1.294	9	1.143	7	0.666	5	0.820	4
	*RPL10*	1.070	1	1.056	5	0.331	1	0.687	1
	*RPS24*	1.210	5	0.871	1	0.599	4	1.307	8
	*28S*	1.722	10	1.345	10	1.109	10	1.554	10
	*SOD*	1.190	4	0.871	1	0.743	9	1.270	7
	*ATP*	1.261	8	1.229	9	0.720	8	1.422	9
	*GAPDH*	1.244	7	1.004	4	0.699	6	1.202	6
	*ACT*	1.096	2	1.100	6	0.527	3	0.833	5
	*a-TUB*	1.224	6	1.180	8	0.709	7	0.752	2
Total	*18S*	1.269	5	1.025	3	0.524	3	0.673	1
	*AK*	1.369	8	1.173	6	0.745	7	0.993	3
	*RPL10*	1.096	1	0.977	1	0.359	1	0.715	2
	*RPS24*	1.369	8	1.291	9	0.752	8	1.491	9
	*28S*	1.750	10	1.414	10	1.168	10	1.722	10
	*SOD*	1.099	2	0.977	1	0.512	2	1.147	6
	*ATP*	1.349	7	1.250	8	0.732	6	1.168	7
	*GAPDH*	1.317	6	1.217	7	0.720	5	1.325	8
	*ACT*	1.128	3	1.064	4	0.532	4	1.065	4
	*a-TUB*	1.262	4	1.136	5	0.757	9	1.097	5

## Data Availability

The data presented in this study are available in the article.

## Supplementary Materials

The following are available online at https://www.mdpi.com/article/10.3390/insects12100902/s1, Figure S1: Amplification specificity of primers in RT-PCR and qRT-PCR.
