# Supplementary figures and images for: Selection and Evaluation of Reference Genes for qRT-PCR in Spodoptera frugiperda (Lepidoptera: Noctuidae)
Source: Insects. 2021 Oct 3;12(10):902. doi: 10.3390/insects12100902 (PMC8538597; doi:10.3390/insects12100902)

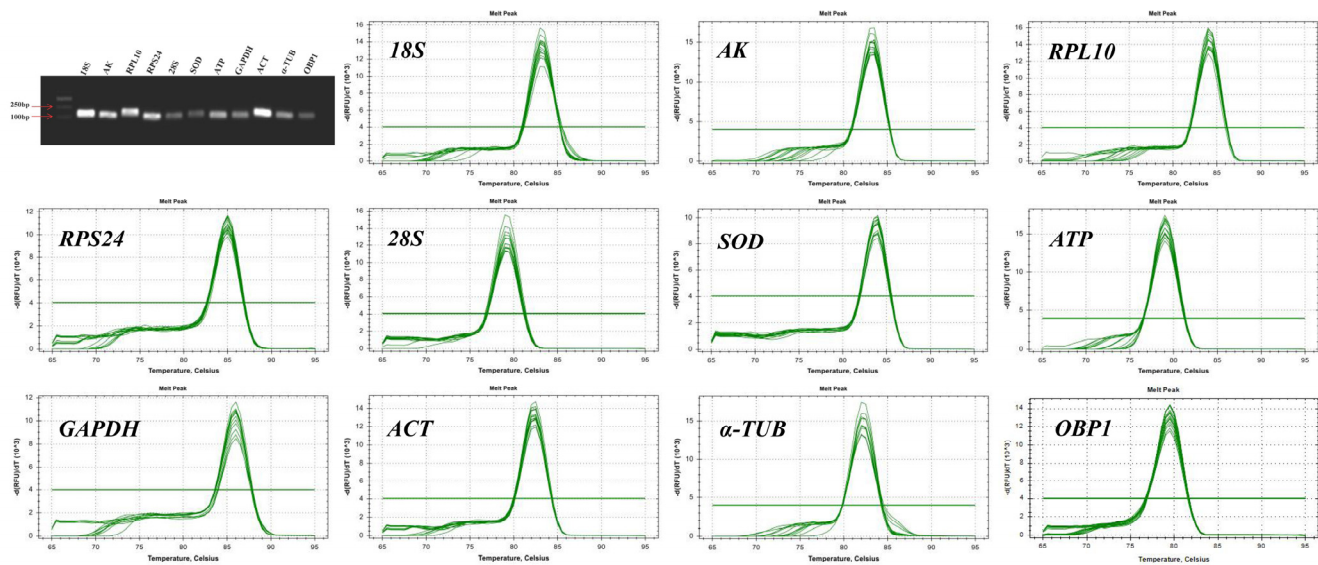

Figure S1. Amplification specificity of primers in RT-PCR and qRT-PCR.

Supplement: Supplementary file 1 [file insects-12-00902-s001.zip › insects-1365804-supplementary.pdf]
